# Reply to Comment on “Distribution of Ixodes scapularis in Northwestern Ontario: Results from Active and Passive Surveillance Activities in the Northwestern Health Unit Catchment Area”

**DOI:** 10.3390/ijerph16112058

**Published:** 2019-06-11

**Authors:** Erin Schillberg, Dorian Lunny, L. Robbin Lindsay, Mark P. Nelder, Curtis Russell, Mike Mackie, Dave Coats, Alex Berry, Kit Ngan Young Hoon

**Affiliations:** 1 Northwestern Health Unit, Kenora, ON P9N 2K4, Canada; eschillberg@nwhu.on.ca (E.S.); mmackie@nwhu.on.ca (M.M.); dcoats@nwhu.on.ca (D.C.); aberry@nwhu.on.ca (A.B.); kyounghoon@nwhu.on.ca (K.N.Y.H.); 2 Canadian Public Health Service, Public Health Agency of Canada, Ottawa, ON K1A 0K9, Canada; 3 Zoonotic Diseases and Special Pathogens, National Microbiology Laboratory, Public Health Agency of Canada, Winnipeg, MB R3E 3R2, Canada; robbin.lindsay@canada.ca; 4 Enteric, Zoonotic and Vector-Borne Diseases, Communicable Diseases, Emergency Preparedness and Response, Public Health Ontario, Toronto, ON M5G 1V2, Canada; Mark.Nelder@oahpp.ca (M.P.N.); Curtis.Russell@oahpp.ca (C.R.)

We thank Mr. Scott for his comments on our 2013 discovery of *Amblyomma cajennense* in northwestern Ontario, through passive surveillance. The tick was collected from a human in August 2013; however, information on travel history and stage are not available. The tick was identified using standard morphological identification keys available in 2013, a year before the work of Nava et al. [1] was available. We did not deposit the tick as a voucher specimen in a collection and realize this was an oversight on our part. We appreciate Mr. Scott’s comment on depositing a voucher specimen and will do so in future work. *A. cajennense* (n = 1) was included for completeness when we presented our passive surveillance results. *A. cajennense* is not mentioned elsewhere in the paper and is not integral to our primary objective of describing *Ixodes scapularis* distribution and *Borrelia burgdorferi* prevalence in northwestern Ontario.

We have acknowledged some of the work that Mr. Scott has performed in this region of northwestern Ontario on ticks and *B. burgdorferi* by citing his study from 2016 [2]. We were also aware of Scott et al. 2017 manuscript [3]; however, because the scope of the testing conducted was much broader than our study we decided not to cite it. However, we would encourage readers to critically review this manuscript and draw their own conclusions about its merit in terms of possible impacts on public health and our understanding of *B. burgdorferi* transmission dynamics.

Mr. Scott believes that blacklegged tick populations were established in northwestern Ontario (the Kenora area specifically) for many years and were just overlooked. In addition, in his opinion, climate change or warming is a non-issue and has not impacted the range expansion of this tick species in Canada. The latter arguments strongly contradict the predictions (e.g., [4,5,6]) validated by empirical observations, which have been published in high quality peer-reviewed journals. This evidence (summarized in [7]) clearly demonstrates that temperature is a key driver (or co-driver) of Lyme disease risk emergence through its impact on vector tick distributions and abundance, and subsequently *B. burgdorferi* transmission cycle occurrence. Empirical data support the notion that climate limits the distribution of blacklegged ticks in Canada and the reality that some regions of the country are too cold to support tick populations. Early field studies on range expansion of blacklegged ticks conducted in Quebec determined that tick populations established in the warmest portions of the province first [8] while additional field surveillance studies in eastern Canada also supported the importance of temperature in determining the spatial pattern of establishment of blacklegged tick populations at a multiprovince geographic scale [9]. Together these data indicate that the spatial pattern of the spread of the tick in Canada, and the geographic limits of its occurrence, are strongly determined by temperature. Leighton et al. [10] provided empirical evidence that during a period of warming, locations in Canada acquired blacklegged tick populations faster the warmer they were, while Ogden et al. [5] and MacPherson et al. [6] showed that the emergence of tick populations in southern Canada was temporally coincident with warming. In our opinion, these studies provide substantive support to the impacts of climate change on blacklegged tick distribution patterns in Canada and we stand by our original statements on this topic.

The attack on the published references in our manuscript is completely unjustified as these studies have all been fully vetted in the peer-reviewed literature and provide empirical data with accompanying statistical analysis to substantiate or support any claims concerning the possible impacts of climate change/warming on the distribution of blacklegged ticks in Canada. It appears that Mr. Scott may not believe that climate change occurs, and he certainly does not think that it has any impact on the distribution of blacklegged ticks. Instead he speculates that public awareness is the primary driver for tick range expansion. According to Mr. Scott, ticks are introduced into Canada by songbirds or deer and wherever there is sufficient snow cover or the photoperiod is permissive, tick populations become established; awaiting eventual discovery by the public. There is plenty of credible scientific evidence that demonstrates that temperature profiles and climate also have a major role to play in determining where and when blacklegged tick populations will establish, and we respectfully disagree with his wholesale dismissal of these impacts.

Although we disagree with Mr. Scott on many of the points he has raised, we, along with public health officials across Canada, take the emergence of Lyme and other tick-borne diseases very seriously and recognize the impact Lyme disease has on Canadians and their families. As such, public health officials continue to work collaboratively to ensure Canadians understand the risk of exposure to infected blacklegged ticks, and that doctors have the knowledge and expertise to diagnose and treat patients as soon as possible after exposure.
